# Artificial Intelligence Legal Literacy and Procedure-Concordant Use of an AI-Enabled Early-Warning System Among Hospital Nurses in Romania

**DOI:** 10.3390/nursrep16100368

**Published:** 2026-10-18

**Authors:** Alina Doina Tănase, Camelia-Oana Mureșan, Ștefania Dinu, Adriana Pădure, Codruța-Eliza Ille, Cristina Ioana Talpoș Niculescu

**Affiliations:** 1 Department of Professional Legislation in Dental Medicine, Faculty of Dental Medicine, “Victor Babeș” University of Medicine and Pharmacy, Eftimie Murgu Square No. 2, 300041 Timișoara, Romania; tanase.alina@umft.ro; 2 Methodological and Research Center Medical Law, Medical Malpractice and Research Ethics, Faculty of Dental Medicine, “Victor Babeș” University of Medicine and Pharmacy, Eftimie Murgu Square 2, 300041 Timișoara, Romania; 3 Discipline of Forensic Medicine, Bioethics, Deontology, and Medical Law, Department of Neuroscience, “Victor Babeș” University of Medicine and Pharmacy, Eftimie Murgu Square 2, 300041 Timișoara, Romania; muresan.camelia@umft.ro; 4 Ethics and Human Identification Research Center, “Victor Babeș” University of Medicine and Pharmacy, Eftimie Murgu Square 2, 300041 Timișoara, Romania; 5 Pediatric Dentistry Research Center (Pedo-Research), Department of Pediatric Dentistry, Faculty of Dental Medicine, “Victor Babeș” University of Medicine and Pharmacy, Eftimie Murgu Square 2, 300041 Timișoara, Romania; 6 Department of Oro-Dental Diagnosis and Ergonomics, Faculty of Dental Medicine, “Victor Babeș” University of Medicine and Pharmacy, Eftimie Murgu Square No. 2, 300041 Timișoara, Romania; adriana.padure@umft.ro (A.P.); ioana.talpos-niculescu@umft.ro (C.I.T.N.); 7 Faculty of Dental Medicine, “Victor Babeș” University of Medicine and Pharmacy, Revoluției Avenue 1989, No. 9, 300580 Timișoara, Romania; ille.codruta@umft.ro; 8 Research Center of Digital and Advanced Technique for Endodontic, Restorative and Prosthetic Treatment (TADERP), “Victor Babeș” University of Medicine and Pharmacy, Revoluției Avenue 1989, No. 9, 300580 Timișoara, Romania

**Keywords:** artificial intelligence, nursing staff, hospital, health literacy, legislation, medical, Romania

## Abstract

**Background and Objectives:** Regulation (EU) 2024/1689, the Artificial Intelligence Act, requires organisations deploying AI systems to ensure appropriate AI literacy and human oversight. We examined whether AI legal literacy was associated with procedure-concordant use of an AI-enabled early-warning system among hospital nurses and whether the association of prior AI-governance training with concordant use persisted after literacy was taken into account. **Materials and Methods:** This single-centre cross-sectional study included 87 nurses in a tertiary university hospital. AI legal literacy was measured with a study-specific 24-item instrument, and procedure-concordant AI use, the primary outcome, was defined as meeting all five criteria of the institutional documentation audit in 10 audited interactions per nurse. The primary analysis was multivariable logistic regression, confirmed in three-level mixed-effects models of 870 audited interactions nested within nurses and wards. Secondary and exploratory analyses (inverse-probability weighting, mediation decomposition, latent class analysis, and an internally validated prediction model) are reported as hypothesis-generating. **Results:** Forty-four nurses (50.6%) were procedure-concordant. Concordance was higher among trained than untrained nurses (70.3% versus 36.0%; *p* = 0.002). Each 10-point increase in AI legal literacy was associated with higher adjusted odds of concordant use (adjusted odds ratio 5.16, 95% CI 2.04–13.06; *p* < 0.001). The association persisted in three-level mixed-effects models (aOR 3.61 per 10 points, 95% CI 1.98–6.58). The association with prior training was attenuated after adjustment for literacy and was not statistically significant after inverse-probability weighting (OR 2.01, 95% CI 0.82–4.90); an exploratory decomposition was consistent with, but could not establish, an indirect association through literacy. **Conclusions:** Higher AI legal literacy was strongly associated with procedure-concordant AI use in this single-centre study. The findings support further evaluation of literacy-focused governance education and institutional oversight in hospital nursing practice; causal and predictive claims await prospective, multicentre confirmation.

## 1. Introduction

Artificial intelligence has moved from methodological promise to routine infrastructure in hospital care, with algorithmic triage, deterioration prediction, and imaging support now embedded in electronic health records rather than confined to research environments [1]. Nursing is the professional group most exposed to this transition, because the systems that have diffused fastest—early-warning scores, sepsis alerts, fall-risk stratification and workload allocation tools—generate outputs that nurses receive first, interpret at the bedside, and act upon within minutes [2]. That exposure coincides with sustained workforce strain across European health systems, where nurse staffing and educational composition remain independently associated with patient outcomes and where digital tools are frequently promoted as a partial remedy for understaffing [3]. The result is a workforce that is simultaneously the principal user of clinical AI and the group least often consulted when such systems are procured, configured, or governed.

Empirical work on how nurses relate to these systems has grown quickly but has concentrated on attitudes and intentions rather than on behaviour. Latent profile analyses of nurses’ attitudes towards AI describe heterogeneous subgroups that differ in optimism, perceived threat, and willingness to engage, with organisational climate emerging as a consistent discriminator [4]. Studies of nursing students and early-career nurses report broadly favourable attitudes coupled with low perceived competence and limited exposure to structured instruction and identify access barriers rather than resistance as the dominant obstacle to adoption [5]. Scoping reviews of AI education programmes for health professionals confirm that most curricula emphasise technical familiarity and clinical use cases, while regulatory, data-protection, and liability content is sparse, unevaluated, or absent [6]. What is measured, in other words, is enthusiasm; what is rarely measured is whether clinicians can lawfully operate the systems they are enthusiastic about.

The regulatory environment has meanwhile changed decisively. Analyses of Regulation (EU) 2024/1689 for health care emphasise that most clinical decision-support software will be classified as high risk, which deployers acquire obligations distinct from those of manufacturers, and that these obligations reach individual professionals through institutional policy [7]. The Regulation itself creates a general AI-literacy duty applicable to all staff who operate or are affected by AI systems and requires deployers of high-risk systems to assign human oversight to natural persons with the necessary competence, authority, and support [8]. The Regulation applies in stages. The AI-literacy duty (Article 4) has applied since 2 February 2025, whereas the deployer obligations for high-risk systems that are medical devices (Article 6(1) and Annex I) were originally scheduled to apply from 2 August 2027, and have since been deferred to 2 August 2028, by Regulation (EU) 2026/1744, the “Digital Omnibus on AI”, adopted on 8 July 2026, and published in the Official Journal on 24 July 2026 [9]. During the period studied here, therefore, the only AI Act duty that applied directly to the hospital was the AI-literacy duty; the oversight, documentation, patient-information, and incident-reporting duties examined in this study arise from the hospital’s own standard operating procedure, adopted in anticipation of the Regulation, and from existing national law, not from directly applicable AI Act obligations. These duties operate alongside, and not instead of, the data-protection framework, under which every clinical inference drawn from patient data constitutes processing of a special category of personal data and must rest on an identifiable lawful basis, with transparency and accountability obligations attaching to the controller [10]. Compliance is therefore no longer an abstract institutional attribute; it is enacted, or not, in individual documentation behaviour.

Two further strands frame the knowledge gap. First, the conformity-assessment literature shows that the boundary between regulated medical device software and unregulated general-purpose tools is poorly understood even by specialists, and that definitional uncertainty propagates from regulators to clinical users [11]. Second, work on the secondary use of health data documents that professionals routinely misidentify the lawful bases and safeguards that apply to data reuse, producing both over-restriction and inadvertent non-compliance [12]. Romania compounds these generic difficulties with a specific profile: national surveys of health professionals show high declared interest in digital tools alongside limited institutional infrastructure, uneven training provision, and persistent uncertainty about the legal status of remotely mediated care [13]. No published study has quantified how well Romanian nursing staff understand the legal architecture that now governs the AI tools already installed on their wards.

The rationale for focusing on nurses specifically, and on legal literacy specifically, follows from where liability actually settles. When a clinician acts on an algorithmic recommendation, the prevailing legal analysis places substantial residual responsibility on the professional who exercised, or failed to exercise, independent judgement, and the safest position is generally to follow standard practice while documenting the reasoning applied to the algorithmic output [14]. That analysis was developed for physician liability in the United States and is applied here only by analogy. In Romania, the nurse’s professional responsibility is defined by the statute governing the nursing profession [15], by the civil-liability provisions for medical personnel in the health-care reform law [16], by the patient-rights law, which establishes the patient’s right to be informed about their care [17], and by the professional code of ethics and deontology [18]; together, these place a personal, non-delegable duty of care, information, and documentation on the individual nurse that an algorithmic recommendation does not displace. Taken together, these sources convert an abstract governance requirement into a set of concrete, auditable documentation behaviours: recording that the output was verified, identifying the tool and version relied upon, noting what the patient was told, escalating discordant outputs through the incident system, and refraining from transferring patient data to unapproved tools. The novelty of the present work is to treat these behaviours as the outcome rather than the assumption, and to test whether they are predicted by measured AI legal literacy rather than by self-reported confidence or training attendance.

Two terms used throughout require definition. We use “AI legal literacy” to denote a clinician’s capacity to recognise the legal and regulatory requirements that apply to an AI system used in care and to interpret and apply those requirements in a concrete clinical situation. “Legal knowledge”, the ability to recall what a rule requires, is a necessary but not sufficient component of that capacity; literacy additionally implies the ability to identify which rule applies in a given situation and what it requires of the nurse. The instrument used in this study (Section 2.3) combines factual-knowledge items with scenario-based application items and therefore measures the knowledge and applied-interpretation components of AI legal literacy. It does not measure the ability to act on that interpretation under the time pressure of a real alert, which can be observed only in practice. In the remainder of the manuscript, “AI legal literacy” refers to the measured score, and “legal knowledge” is used only where the factual component alone is meant.

We therefore designed a cross-sectional study of nursing staff in a Romanian tertiary hospital in which an AI-enabled early-warning module is used across all adult inpatient areas. The primary objective was to estimate the prevalence of procedure-concordant AI use and to quantify its association with AI legal literacy. Secondary and exploratory objectives, reported as hypothesis-generating, were to compare nurses with and without prior AI-governance training across literacy, attitudinal, and behavioural outcomes; to identify latent profiles of governance engagement; to explore, within an explicitly associational framework, whether the association between training and concordant use is consistent with an indirect pathway through legal literacy; and to develop and internally validate an exploratory literacy-based model, reported according to TRIPOD+AI, to examine whether it has sufficient discrimination, calibration, and net benefit to warrant prospective evaluation as a tool for targeting remediation.

## 2. Materials and Methods

### 2.1. Study Design, Setting, and Data Sources

This is a single-centre, analytical cross-sectional study of hospital nursing staff, reported in accordance with the Strengthening the Reporting of Observational Studies in Epidemiology (STROBE) recommendations for cross-sectional designs [19]. The study was conducted in a 1050-bed tertiary university emergency clinical hospital in Western Romania that deployed an AI-enabled early-warning module (AI-EWS) inside its electronic health record in September 2024, 18 months before the survey window. The module continuously scores adult inpatients for physiological deterioration and pushes prioritised alerts to the ward nursing station; it does not prescribe treatment. The module is a commercial software product whose intended purpose is the continuous monitoring of adult inpatients’ recorded vital signs and the flagging of a risk of clinical deterioration for prioritised clinical review; it is CE-marked as a class IIa medical device under Regulation (EU) 2017/745 (rule 11) [20]. As an AI system that is a product subject to third-party conformity assessment under Annex I of the AI Act, it is expected to be treated as a high-risk AI system under Article 6(1), but the corresponding deployer obligations were not yet applicable during the study period (Section 1). The hospital nevertheless classifies the module internally as high risk and governs its use through a local standard operating procedure (SOP), adopted in September 2024, that specifies human-oversight, documentation, patient-information, incident-reporting, and data-transfer duties. The five audit criteria used in this study (Section 2.3) are the duties of that SOP; they are institutional requirements informed by the AI Act, the General Data Protection Regulation (GDPR), and national law, not obligations that the AI Act itself imposed on individual nurses at the time of the study. All adult inpatient areas use the same module, the same alert thresholds, and the same documentation template, so exposure to the technology is uniform across clinical areas. Two features of the deployment define the sampling frame. First, under the SOP, alerts are acknowledged and documented by nurses who hold the AI-EWS alert-response role in the electronic health record; the hospital assigns this role to a limited number of nurses per ward (typically shift coordinators and senior staff nurses) after module authorisation, and other nurses view alerts but do not document the response. Second, the institutional AI-EWS documentation-audit programme, from which the audited interactions were drawn, was operating in 16 wards during the study period as the first phase of a staged roll-out. The sampling frame therefore comprised the nurses holding the alert-response role on these 16 wards, and the study is a census of that frame rather than of all nursing staff in the hospital. The participating nurses were employed on 16 wards nested within the four clinical areas (three intensive-care units, two emergency-department areas, five surgical wards, and six medical wards; median: five nurses per ward; range: 3–9); the ward was recorded for every participant and every audited interaction so that the hierarchical structure of the data could be accommodated in the analysis (Section 2.4).

The study followed a fixed sequence. Ethics approval was granted on 26 January 2026. The audit window comprised the three calendar months from 1 December 2025 to 28 February 2026, during which the AI-EWS interactions that were later audited took place; the staff was unaware of the study during this window, although the institutional audit programme itself was known to them. Questionnaires were completed between 2 March and 3 April 2026, after the audit window had closed, so that completing the questionnaire could not influence the audited behaviour. Retrieval of audited records, electronic health record audit trails and monitoring-log extracts, and the documentation audit itself took place between 6 April and 15 May 2026, after questionnaire completion; no study data were accessed before ethics approval. Because literacy was measured after the behaviour with which it is associated, the design cannot establish that literacy preceded behaviour (Section 4.2).

The hospital is a clinical teaching site of the authors’ university. None of the authors are employed by the hospital’s nursing directorate, clinical-governance department, or information-security function. None were involved in procuring or configuring the AI-EWS or in delivering the governance training, and the hospital had no role in the design, analysis, or reporting of the study. Access to data was governed by a research agreement between the hospital and the university.

The analysis used participant-level data collected for this study from the questionnaire, institutional training records, documentation audit, and the demographic and employment sources described below. Data preparation included consistency checks, coding of the pre-specified variables, and handling of the small amount of missing information described in Section 2.3. All reported statistics, tables, and figures were calculated from the study dataset using the analysis procedures specified in Section 2.4.

### 2.2. Eligibility Criteria and Group Allocation

Eligible participants were registered nurses and licensed practical nurses employed on a permanent or fixed-term contract in an adult inpatient area of the study hospital, with at least 6 months of continuous service in that area and at least one documented AI-EWS interaction in the 3 months preceding the survey window. Nurses working exclusively in outpatient clinics, day-case units, paediatrics, obstetrics, or the operating theatre complex were excluded because the AI-EWS is not deployed there. Also excluded were nurses on long-term leave for the whole of the audit window, agency staff without a hospital electronic health record account, and staff whose documentation could not be linked to a single primary clinical area. Of the 311 nurses employed on the 16 wards during the audit window, 138 held the alert-response role and were assessed for eligibility; 42 were excluded (fewer than 6 months of service in the area, 9; no documented AI-EWS interaction in the audit window, 17; long-term leave, 7; agency staff without a hospital record account, 3; documentation not linkable to a single primary area, 6). Ninety-six nurses satisfied the eligibility criteria; six declined and three did not return the questionnaire, so 87 provided complete consent and questionnaire data, corresponding to a participation fraction of 90.6%. The analysis population is therefore all 87 participants.

Group allocation was observational rather than randomised. The exposure of interest was prior structured training in AI governance and health-data protection, defined as documented attendance at an accredited course of at least 4 contact hours covering the AI Act, the General Data Protection Regulation, and institutional AI policy, completed in the 24 months preceding the survey. Attendance was verified against the hospital training register rather than self-reported, and nurses whose attendance record was ambiguous were classified as untrained. Thirty-seven nurses (42.5%) were classified as trained and 50 (57.5%) as untrained. Clinical area (intensive care, emergency, surgical, medical) was treated as a pre-specified stratification variable for descriptive and subgroup analyses. In the regression models, it was entered as intensive care versus other wards, a contrast chosen before analysis because intensive-care units have a distinct alert burden and workflow, and because a four-level variable would have consumed three parameters with only 44 outcome events; a sensitivity analysis using the four-level variable is reported in Section 3.7. In addition, the number of ward-level governance enablers present—a designated AI champion, a visible standard operating procedure at the workstation, superuser training, and periodic audit feedback—was recorded as an ordinal contextual variable ranging from zero to four. Ward-level enablers were ascertained from the clinical-governance department’s records and confirmed with the ward manager; periodic audit feedback at ward level meant that the ward received documentation-audit results at least quarterly. Two nurse-level self-reported indicators used in the analyses are defined here: “receives periodic audit feedback” denotes that the nurse reported having personally received individual or ward-level feedback on AI-EWS documentation at least once in the preceding six months, and “regular use of AI-enabled tools” denotes self-reported use of any hospital-approved AI-enabled tool, other than passive receipt of AI-EWS alerts, at least weekly during the preceding month.

### 2.3. Variables, Outcomes, and Data Collection

The primary outcome was procedure-concordant AI use, a binary variable defined a priori as satisfying all five criteria of a documentation audit applied to a random sample of 10 AI-EWS interactions per nurse. The five criteria were: documented human verification of the AI output before acting on it; recording of the AI tool identifier and software version in the nursing note; a note that the patient or proxy was informed that an AI-assisted assessment contributed to the care decision, where the local procedure required it; reporting of any discordant or clinically implausible AI output through the institutional incident system; and absence of any transfer of identifiable patient data to an AI tool not approved by the hospital. A nurse was classified as concordant only if all five criteria were met in every audited interaction, which is a deliberately conservative definition. The outcome therefore measures concordance with the hospital’s own SOP, a specific institutional governance framework, and not a universally established standard of legally or clinically appropriate AI use; this matters for generalisability (Section 4.2). Because the composite requires every criterion to be met in every interaction, it is stringent, and the count of criteria met and the sensitivity analyses described below were pre-specified to show that the main association does not depend on this stringency. The nurse-level binary outcome was pre-specified because the unit at which the institutional standard operating procedure assigns responsibility, and at which remediation is delivered, is the individual nurse; the count of criteria met and interaction-level analyses (Section 2.4) were retained to preserve the information contained in the 10 audited interactions per nurse. Pre-specified secondary outcomes were the number of criteria met (0–5), the AI legal literacy score, attitude towards clinical AI, perceived legal risk, digital self-efficacy, and four binary governance indicators covering awareness of the AI Act, awareness of the institutional standard operating procedure, routine consent notation, and routine reporting of discordant output. The four indicators were self-reported yes/no questionnaire items (for example, “I routinely record in the nursing note that the patient was informed that an AI-assisted assessment contributed to the decision”) and are therefore self-reported habits rather than audited behaviour. Two of them, routine consent notation and routine reporting of discordant output, overlap in content with audit criteria three and four, which is taken into account when the latent class analysis is interpreted (Section 3.8).

Two of the five criteria are conditional. The patient-information criterion applied only to interactions in which the local procedure required disclosure, namely when the AI-assisted assessment contributed to a change in the care plan (escalation to medical review, transfer, or a change in monitoring frequency); the incident-reporting criterion applied only to interactions in which the audited record showed a discordant or clinically implausible output, defined as an alert generated, while all recorded vital signs were within the ward’s normal range or an output that the nurse’s own note described as inconsistent with the clinical picture. Conditional criteria were scored at interaction level as met, not met, or not applicable. In the primary analysis, a nurse was classified as satisfying a conditional criterion when it was met in every applicable interaction, or when no applicable interaction occurred, because the absence of a triggering event means that no obligation was breached; this is the same convention used in the institutional audit programme. Because this convention can favour nurses who happened to have few triggering events, two pre-specified sensitivity analyses were performed: (i) the primary analysis was repeated in the subset of nurses with at least one applicable interaction for both conditional criteria, and (ii) the outcome was redefined using only the three unconditional criteria. The number of applicable interactions per nurse was recorded for both conditional criteria.

The audit was performed by two trained auditors, a quality-and-patient-safety nurse from the hospital’s clinical-governance department and a research nurse from the university, using a standardised audit form with written scoring rules for each criterion. For each nurse, all AI-EWS interactions in the audit window were listed from the module’s alert log, and 10 were selected with computer-generated random numbers (seed recorded in the analysis code). The auditors had access only to the electronic health record and were blinded to training status, questionnaire responses, and literacy scores, which were held by the university team and linked to the audit results only after the audit was complete. A random 20% subsample of nurses (18 nurses, 180 interactions) was double-coded independently; agreement was κ = 0.86 for human verification, 0.94 for tool identifier and version, 0.82 for patient information, 0.84 for incident reporting, and 0.91 for interaction-level concordance. Disagreements were resolved by consensus with a third reviewer before the analysis dataset was locked.

The fifth criterion, absence of any transfer of identifiable patient data to an AI tool not approved by the hospital, cannot be verified from the nursing record alone. It was therefore assessed from three linked sources for the audit window: (i) the content of the audited nursing notes, which were screened for text or output attributed to a tool outside the approved list; (ii) the hospital’s data-loss-prevention and web-proxy logs, which record copy, export, and upload events from the electronic health record session and access to external generative-AI domains from clinical workstations, linked to the user account of the nurse; and (iii) the electronic health record audit trail of data exports. A nurse was classified as not meeting the criterion if any source showed a transfer of identifiable data to an unapproved tool during the audit window. Because the monitoring logs relate to the audit window as a whole rather than to individual interactions, this criterion was assigned at nurse level: in the interaction-level and multilevel analyses, all 10 audited interactions of a nurse who did not meet the criterion were classified as non-concordant, which is the conservative convention of the institutional audit, and a sensitivity analysis in which interaction-level concordance was defined using only the four record-based criteria is reported in Section 3.10. The criterion is therefore strictly one of no detected transfer: it cannot capture transfers made from personal devices or outside the hospital network, and its sensitivity depends on the coverage of the institutional monitoring systems, which is acknowledged as a limitation.

The data-loss-prevention and web-proxy logs are maintained by the hospital’s information-security function under its information-security policy, of which all staff are informed at induction and annually, and the hospital already uses them for its routine AI-EWS audit; the study therefore introduced no monitoring that did not previously exist. The information sheet and consent form stated explicitly that the hospital’s data protection officer would extract, for the audit window, a single binary indicator (any detected transfer of identifiable patient data to an unapproved tool: yes or no) linked to the participant’s study code, and that no log content would be transferred to the research team. The key linking study codes to identities was held by the university team only; the hospital received no participant-level research results and could not learn from the study which participants had or had not met any criterion. The transfers detected during the audit window had already been identified by the hospital’s routine monitoring, independently of the study, and were handled under its existing data-breach procedure (Section 3.2).

AI legal literacy was measured with a 24-item instrument developed de novo for this study because no validated instrument covering the AI Act, the GDPR, and institutional AI governance for nurses was available. The instrument covers five domains—AI Act risk classification, GDPR lawful basis for processing, patient information and consent, incident and serious-incident reporting duties, and professional liability and human oversight—scored as the percentage of correct responses and reported on a 0–100 scale, with domain subscores on the same scale. Items were drafted by two authors with expertise in medical law and one with expertise in nursing from the text of the AI Act, the GDPR, the national patient-rights and nursing-practice legislation, and the institutional SOP. Each item is a four-option multiple-choice question with one correct answer. Fourteen items test factual knowledge (for example, which lawful basis permits the processing of health data for care), and 10 are scenario-based application items in which a short ward vignette is presented and the respondent must identify what the applicable rule requires of the nurse (for example, whether an alert that leads to escalation must be disclosed to the patient). The items are distributed across the domains as follows: AI Act risk classification, 5; GDPR lawful basis, 5; patient information and consent, 5; incident and serious-incident reporting, 4; professional liability and human oversight, 5. Content validity was assessed by a seven-member panel (two health lawyers, two nurse educators, a hospital data protection officer, a clinical informatician, and a nurse manager) who rated the relevance of each of an initial pool of 32 items on a four-point scale; items with an item-level content validity index below 0.78 were removed or revised, and the 24 retained items had item-level indices of 0.86–1.00 and a scale-level index (averaging method) of 0.93 [21]. The instrument was pilot-tested in 15 nurses from a paediatric ward not included in the study, which led to the rewording of three items. It was administered in Romanian, on paper, under supervision and without access to reference material. Items were scored as correct or incorrect without partial credit. The full instrument, in Romanian with an English translation and the answer key, is available from the corresponding author on request. Attitude, perceived legal risk, perceived organisational support, and digital self-efficacy were captured on 0–100 visual analogue scales. Demographic, employment, and workload variables were extracted from the hospital human-resources record. Missing values were confined to three variables—perceived organisational support (4 participants, 4.6%), patients per shift (3, 3.4%) and night shifts per month (2, 2.3%)—with no missing values in the primary outcome, the exposure or the literacy score.

In the study sample, item difficulty (proportion correct) ranged from 0.21 to 0.78 and corrected item–total point-biserial correlations from 0.28 to 0.62. Internal consistency at item level was KR-20 = 0.89; a Cronbach’s α calculated across the five domain scores (0.93; Spearman–Brown split-half coefficient 0.94; corrected domain–total correlations 0.80–0.84) is also given for comparison, although it overstates item-level consistency. Because the domain subscores were highly inter-correlated, dimensionality was examined with an exploratory factor analysis of the tetrachoric correlation matrix and a one-factor confirmatory model. The first factor explained 46% of the common variance with a first-to-second eigenvalue ratio of 4.8, and the one-factor model fitted adequately (comparative fit index 0.95, Tucker–Lewis index 0.94, root mean square error of approximation 0.048), whereas a five-factor model did not improve fit (change in comparative fit index < 0.01) and yielded inter-factor correlations of 0.82–0.91. The instrument is therefore treated as essentially unidimensional: the total score is used in all models, and the domain subscores are reported descriptively only. With 87 participants, these dimensionality analyses are approximate and preliminary. Evidence of construct validity is limited to known-group differences (Section 3.2). The instrument is study-specific and has not been validated externally; external validation is a requirement for future work.

### 2.4. Statistical Analysis

Given the sample size (87 nurses, 44 outcome events), the analyses are organised in three tiers. The primary analysis is the multivariable logistic regression of procedure-concordant use on AI legal literacy (Model 2), confirmed in three-level mixed-effects models of the 870 audited interactions. Pre-specified secondary analyses are the comparison of trained and untrained nurses, the univariable screen, the robustness analyses of the training association (inverse-probability weighting, multiple imputation and the E-value), and the restricted cubic spline. All other analyses, namely the subgroup and effect-modification analyses, the correlation matrix, the latent class analysis, the mediation decomposition, and the evaluation of the illustrative prediction model, are exploratory and hypothesis-generating; only their principal findings are reported.

Continuous variables are summarised as mean ± standard deviation and categorical variables as counts and percentages. Normality was assessed with the Shapiro–Wilk test in each comparison group and homogeneity of variance with Levene’s test. Two-group comparisons of continuous variables used Welch’s *t* test when both groups satisfied the normality criterion and the Mann–Whitney U test otherwise. Four-group comparisons used one-way analysis of variance or the Kruskal–Wallis test under the same rule, with Dunn’s post-hoc procedure and Holm adjustment. Because selecting tests on the basis of preliminary normality testing is debated, every continuous comparison was repeated with Welch’s *t* test (or analysis of variance) and with the distribution-free alternative irrespective of normality; no inference changed, and the tables report the pre-specified rule. Because of the modest sample size, all 2 × 2 categorical comparisons used Fisher’s exact test, and all larger contingency tables used the Fisher–Freeman–Halton exact test with a Monte Carlo *p*-value based on 20,000 replicates. Effect sizes are reported as Hedges’ g for continuous contrasts, Cramér’s V for categorical contrasts, η^2^ or ε^2^ for four-group comparisons, and standardised mean differences for baseline balance, with values below 0.10 taken to indicate negligible imbalance. Binomial proportions are accompanied by Wilson score intervals. Correlations are Spearman rank coefficients with Fisher z-transformed 95% confidence intervals. Two-sided *p*-values are reported exactly to three decimal places, with *p* < 0.001 used below that threshold, and α was set at 0.05; the Benjamini–Hochberg procedure controlled the false-discovery rate across the univariable screen and the domain-level comparisons.

Multivariable logistic regression modelled procedure-concordant AI use with a pre-specified adjustment set comprising age, sex, nursing qualification, clinical area, and ward governance enablers; a second model added AI legal literacy. The adjustment set was chosen from a directed acyclic graph in which qualification, age, sex, clinical area, and ward governance enablers are treated as common causes of training, literacy, and documentation behaviour. Model 1 estimates the total association of training with concordance. Model 2 estimates the association of literacy with concordance conditional on training; because literacy is a plausible consequence of training, Model 2 conditions on a potential mediator of the training association, so the attenuation of the training coefficient between Model 1 and Model 2 is expected by design and is not evidence that training is unimportant. Attitude towards AI, digital self-efficacy, and perceived legal risk were not included in the pre-specified models because they may themselves be influenced by training and literacy; a sensitivity model that additionally adjusts for attitude, self-efficacy, and English proficiency is reported in Section 3.7. The interaction between literacy and perceived organisational support was not pre-specified; it was examined post-hoc, and its *p*-value is reported with a Bonferroni adjustment for the eight interaction tests performed. Multicollinearity was assessed with variance inflation factors, model fit with the likelihood-ratio test, McFadden’s pseudo-R^2^ and the Hosmer–Lemeshow test using 10 groups of predicted risk (8 degrees of freedom) for all models, and discrimination with the area under the receiver operating characteristic curve. Latent class analysis was fitted by expectation–maximisation to six binary governance indicators, with 25 random starts per solution, and the number of classes was selected using the Bayesian information criterion, entropy, smallest-class size, and a bootstrap likelihood-ratio test with 99 replicates. An exploratory mediation analysis of the pathway from training through AI legal literacy to concordant use used the quasi-Bayesian Monte Carlo approximation with 5000 simulations, reporting the indirect (average mediation), direct, and total effects on the probability scale. Because the design is cross-sectional and training was not randomised, the identifying assumptions of causal mediation (no unmeasured exposure–outcome, mediator–outcome or exposure–mediator confounding, and temporal precedence of training over literacy) cannot be verified; the analysis is therefore presented as a descriptive decomposition of an association and not as evidence of a causal mechanism, and the proportion mediated is not reported because it is unstable when the direct-effect estimate is close to zero or of opposite sign. Robustness was examined with stabilised inverse-probability-of-treatment weights truncated at the first and 99th percentiles, with multiple imputation by chained equations (20 imputations, Rubin’s rules) for the missing-data sensitivity analysis, and with the E-value for unmeasured confounding. The pre-specified propensity model included age, sex, qualification, intensive-care assignment, ward governance enablers, English proficiency, and digital self-efficacy. Because self-efficacy may be influenced by training, and because attitude towards AI showed the largest baseline imbalance, a re-specified propensity model that removed self-efficacy and added attitude towards AI and nursing experience was fitted post-hoc and is reported alongside the original. Multiple imputation is reported in two forms: with the Model 1 covariates only, as the missing-data sensitivity analysis, and with perceived organisational support and perceived legal risk added, as a separate extended-adjustment model. The E-value was calculated from the weighted risk ratio as RR + √[RR(RR − 1)], using the risk ratio rather than the odds ratio because the outcome is common, and is reported for the point estimate and for the confidence limit closest to the null [22]. Covariate balance after weighting was assessed with standardised mean differences, and residual imbalance is reported. Dose–response was modelled with restricted cubic splines with four knots at the 5th, 35th, 65th, and 95th centiles and a likelihood-ratio test for non-linearity. Internal validity was assessed by bootstrap optimism correction (1000 replicates) of the area under the curve and the calibration slope, together with the Brier score and decision-curve analysis across the pre-specified threshold probabilities. The 95% confidence interval for the optimism-corrected area under the curve was obtained from the 2.5th and 97.5th percentiles of the bootstrap distribution of the apparent area, shifted by the mean optimism. Net benefit is reported both as apparent and as bootstrap-corrected (the apparent net benefit minus the mean optimism estimated over the same 1000 replicates). Analyses used Python 3.11 (Python Software Foundation, Wilmington, DE, USA) with NumPy, SciPy, pandas, statsmodels, and scikit-learn.

Because audited interactions are nested within nurses and nurses are nested within wards, the nurse-level analyses were complemented by pre-specified multilevel sensitivity analyses that use the full hierarchical structure. First, at the level of the 870 audited interactions (10 per nurse), interaction-level concordance (all five criteria met, with conditional criteria scored as described in Section 2.3) was modelled with a three-level mixed-effects logistic regression with random intercepts for nurse and for ward, fitted by adaptive Gauss–Hermite quadrature with the same fixed effects as Model 2; variance components are reported with intraclass correlation coefficients on the latent (logistic) scale, where the nurse-level coefficient is (σ^2^nurse + σ^2^ward)/(σ^2^nurse + σ^2^ward + π^2^/3), the correlation between two interactions of the same nurse, and the ward-level coefficient is σ^2^ward/(σ^2^nurse + σ^2^ward + π^2^/3), and the contribution of each level was tested with likelihood-ratio tests against models omitting the corresponding random intercept. Second, the interaction-level model was refitted by generalised estimating equations with an exchangeable working correlation within nurses and cluster-robust standard errors, which estimates population-averaged rather than nurse-specific associations. Third, the nurse-level Model 2 was refitted with a random intercept for wards, and separately with cluster-robust standard errors for wards, to examine whether ward-level clustering altered the nurse-level inference. Mixed-effects and generalised-estimating-equation models were fitted in statsmodels (BinomialBayesMixedGLM and GEE) and checked in R 4.3 (R Foundation for Statistical Computing, Vienna, Austria; lme4 1.1-35 and geepack 1.3.9).

### 2.5. Prediction-Model Development, Sample-Size Rationale, and Intended Use

Model 2 was additionally evaluated, for illustrative purposes only, as an exploratory prediction model and is reported according to the TRIPOD+AI statement [23]. The sample size was fixed by the census of the eligible population (96 eligible nurses, of whom 87 participated) and was not determined by the requirements of model development. A power calculation performed before data collection, based on the expected size of the eligible frame and an expected concordance of 50%, indicated that a sample of 87 nurses would have more than 80% power at a two-sided α of 0.05 to detect an odds ratio of 2.0 per standard deviation of AI legal literacy. For prediction-model development, the criteria of Riley et al. [24] applied post-hoc (seven candidate parameters, outcome prevalence 0.506, and an anticipated Cox–Snell R^2^ of 0.39 corresponding to the observed likelihood-ratio statistic) indicate a minimum of 384 participants, driven by the requirement for a precise intercept, with 106–123 participants needed to keep expected shrinkage above 0.90 and the optimism in apparent R^2^ below 0.05; the available sample of 87 nurses with 44 events (6.3 events per parameter) therefore falls short of these targets. The model is accordingly presented as hypothesis-generating, its bootstrap-corrected calibration slope is reported as a direct estimate of overfitting, and it is not proposed for implementation before external validation. A literacy-only model, with the literacy score as the sole predictor, was evaluated in the same way for comparison. The complete model specification (intercept, regression coefficients, and standard errors on the log-odds scale) and the full analysis code (Python and R scripts for data preparation, all models, bootstrap validation and decision-curve analysis) are available from the corresponding author on request (see Data Availability Statement).

The intended use of the model is to prioritise nurses for a targeted remediation intervention that is already part of the institutional governance programme: a 2-h, case-based refresher module on the AI Act, GDPR lawful basis, patient information, incident reporting, and human-oversight documentation, followed by one-to-one review of the nurse’s own audited interactions with the ward AI champion within 4 weeks. The institutional audit identifies non-concordant nurses directly, but it is resource-intensive (approximately 45 min of auditor time per nurse) and is run once a year on each ward; a brief literacy test could, if validated, flag nurses for remediation between audit cycles or on wards not yet covered by the audit. Whether that is useful is a question for prospective study, not one that the present analysis can answer. Because remediation is directed at nurses who are unlikely to be concordant, decision-curve analysis [25] was performed on the complementary prediction, the probability of non-concordant use (1 minus the predicted probability of concordance), so that “treat” corresponds to offering remediation and “treat all” to remediating every nurse. The consequences of the two errors were specified before the analysis: an unnecessary remediation costs approximately 3 hours of nurse and champion time and carries a minor risk of disengagement, whereas a missed remediation leaves a nurse practising non-concordantly, with continuing regulatory exposure for the hospital and potential patient-safety consequences. In consultation with the hospital’s clinical governance committee, a missed remediation was judged two to four times more harmful than an unnecessary one, which corresponds to threshold probabilities of non-concordance between 20% and 33%; net benefit is reported for this pre-specified range only. A nurse whose predicted probability of non-concordance exceeds the chosen threshold would be offered remediation. The net reduction in unnecessary remediations per 1000 nurses screened, compared with remediating everyone, is reported at each threshold.

## 3. Results

### 3.1. Characteristics of the Study Population

Of the 311 nurses employed on the 16 wards, 87 were analysed (Section 2.2). Table 1 summarises the characteristics of the 87 participating nurses overall and according to prior AI-governance training.

Among the 87 participants, 72 (82.8%) were female and the mean age was 38.7 ± 9.9 years. Thirty-seven (42.5%) had prior AI-governance training. The training groups were similar in age, nursing experience, clinical area, night-shift burden, patients per shift, and AI-EWS alerts reviewed, while differences were observed in nursing qualification, ward governance enablers, audit feedback, digital self-efficacy, attitudes towards clinical AI, and perceived organisational support (Table 1).

### 3.2. Primary and Secondary Outcomes

The primary and secondary outcomes, overall and by training status, are presented in Table 2, and the domain-level structure of AI legal literacy is displayed in Figure 1.

Procedure-concordant AI use was observed in 44/87 nurses (50.6%). Concordance was higher among trained than untrained nurses (70.3% versus 36.0%; OR 4.20, 95% CI 1.69–10.45; *p* = 0.002). Trained nurses also had higher AI legal literacy, attitudes towards clinical AI, digital self-efficacy, awareness of the institutional standard operating procedure, routine consent notation, and routine reporting of discordant outputs, while perceived legal risk was lower (Table 2). Known-groups differences supported the construct validity of the literacy instrument: scores were higher in trained nurses (g = 1.50) and in nurses with a bachelor’s or master’s qualification than in post-secondary graduates (52.1 ± 11.4 versus 37.8 ± 8.3; g = 1.35) and correlated with awareness of the AI Act (ρ = 0.41, *p* < 0.001).

Applicability of the two conditional criteria was recorded for every audited interaction. The patient–information criterion applied to 312 of the 870 interactions (35.9%), involving 79 nurses (90.8%). Of these, 62 (78.5%) met the criterion in every applicable interaction; together with the 8 nurses who had no applicable interaction, 70 nurses (80.5%) satisfied it (Table 3). The incident-reporting criterion applied to 148 interactions (17.0%), involving 61 nurses (70.1%). Of these, 38 (62.3%) reported every discordant output; together with the 26 nurses who had no discordant output, 64 nurses (73.6%) satisfied it. No nurse was classified as concordant solely because both conditional criteria were inapplicable: all 44 concordant nurses had at least one applicable interaction for at least one conditional criterion. Six nurses (6.9%) did not meet the data-transfer criterion. Four were identified from data-loss-prevention or web-proxy logs showing an upload from the electronic health record session to an unapproved generative-AI domain, and two from nursing notes containing output attributed to an unapproved tool; the export audit trail identified no additional cases. All six transfers had been detected by the hospital’s routine monitoring before the study linked them to participants. Each was assessed by the data protection officer as a personal-data breach under Article 33 of the GDPR and recorded in the breach register. Two were notified to the national supervisory authority (Autoritatea Națională de Supraveghere a Prelucrării Datelor cu Caracter Personal); four were documented without notification because the uploaded content was judged unlikely to put the patients concerned at risk. The nurses involved received the non-punitive remediation provided for in the SOP, and no disciplinary action resulted; the research team did not identify any participant to the hospital. The main association did not depend on the scoring of the conditional criteria. Among the 56 nurses with at least one applicable interaction for both conditional criteria, 26 (46.4%) were concordant, and the adjusted literacy association was materially unchanged (aOR 4.72 per 10 points, 95% CI 1.63–13.7; *p* = 0.004). When only the three unconditional criteria were used, 56 nurses (64.4%) were concordant and the association remained (aOR 4.38 per 10 points, 95% CI 1.85–10.4; *p* = 0.001).

Across all five AI legal literacy domains, mean scores were higher in trained nurses, with between-group differences of 14.8–16.3 points and Benjamini–Hochberg-adjusted q-values < 0.001. AI Act risk classification had the lowest mean score in both groups (Figure 1).

### 3.3. Documentation Criteria and AI Legal Literacy Across Clinical Areas

Table 3 disaggregates the five audit criteria and the principal continuous measures by clinical area, and Figure 2 examines the contribution of ward-level governance infrastructure.

Across clinical areas, recording the AI tool identifier and version differed significantly (*p* = 0.001), as did avoidance of unapproved AI tools (*p* = 0.041). Procedure-concordant use, total criteria met, AI legal literacy, perceived legal risk, and governance-enabler counts did not differ significantly by clinical area (Table 3). Observed concordance increased from 22.2% with no ward governance enablers to 83.3% with all four; the unadjusted trend was significant (*p* = 0.037). After adjustment (Model 2; Section 3.7), the association per additional enabler was no longer significant (aOR 1.32, 95% CI 0.77–2.24; *p* = 0.309; Figure 2).

### 3.4. Subgroup Analyses and Effect Modification

The association between prior training and procedure-concordant AI use within pre-specified subgroups and a post-hoc analysis of effect modification by perceived organisational support are both exploratory and are summarised here only briefly.

Training was associated with higher concordance in the same direction across all pre-specified subgroups. None of the interaction tests were statistically significant, with interaction *p*-values ranging from 0.515 to 0.886. In a post-hoc analysis of the 83 participants with complete organisational-support data, a literacy-by-support interaction was observed (unadjusted likelihood-ratio *p* = 0.028; Bonferroni-adjusted *p* = 0.22 for the eight interaction tests performed). The estimated association of a 10-point literacy increase with concordant use was larger at lower organisational-support values and smaller at higher support values. This interaction was not pre-specified, does not survive adjustment for multiple testing, and is hypothesis-generating only.

### 3.5. Univariable Associations with Procedure-Concordant AI Use

Table 4 presents unadjusted odds ratios for every candidate correlate of the primary outcome, together with false-discovery-rate-adjusted q-values.

AI legal literacy had the strongest univariable association with concordant use (OR 5.47 per 10 points; q < 0.001). Nursing qualification, prior training, awareness of the AI Act and institutional standard operating procedure, regular AI-tool use, attitude, digital self-efficacy, and perceived legal risk also remained significant after false-discovery-rate adjustment; workload and alert-volume variables were not significant (Table 4).

### 3.6. Correlation Structure of the Continuous Measures

Spearman rank correlations were calculated among the nine continuous measures.

The number of audit criteria met correlated most strongly with AI legal literacy (ρ = 0.671; *p* < 0.001). Smaller positive correlations were observed with attitude, digital self-efficacy, perceived organisational support, and ward governance enablers, while perceived legal risk was inversely correlated; AI-EWS alert volume showed no significant correlation with literacy or audit criteria met.

### 3.7. Multivariable Models

Table 5 reports two nested multivariable logistic-regression models, and Figure 3 displays the dose–response relationship between AI legal literacy and the adjusted probability of procedure-concordant AI use.

Before AI legal literacy was added, prior training and higher nursing qualification were independently associated with concordant use. In Model 2, AI legal literacy was strongly associated with concordance (aOR 5.16 per 10 points; *p* < 0.001). The training and qualification coefficients were attenuated and no longer significant, as expected when a potential mediator is conditioned on (Section 2.4). Discrimination increased from an AUC of 0.787 to 0.875, and all variance inflation factors were ≤ 2.00 (Table 5). In sensitivity models, the literacy association was similar when attitude towards AI, digital self-efficacy, and English proficiency was added to Model 2 (aOR 4.41 per 10 points, 95% CI 1.63–11.9; *p* = 0.003), when clinical area was entered as the four-level variable (aOR 5.02, 95% CI 1.95–12.9; *p* = 0.001) and in the literacy-only model (OR 5.47, 95% CI 2.59–11.56; Table 4).

The adjusted probability of concordant use increased across the observed AI legal literacy range. The overall literacy association was significant (*p* < 0.001), and the spline did not show significant non-linearity (*p* = 0.420), supporting the linear specification used in Model 2. Uncertainty widened at the extremes of the observed score distribution (Figure 3).

### 3.8. Latent Classes of AI-Governance Engagement

Latent class analysis of six binary governance indicators was performed, and the resulting classes were characterised.

Model selection favoured a two-class solution. The governance-engaged class included 27 nurses and had higher AI legal literacy, more prior training, and more procedure-concordant use than the limited-engagement class; concordant use was 70.4% versus 41.7% (OR 3.32, 95% CI 1.26–8.79; *p* = 0.015). Two of the six indicators (routine consent notation and routine reporting of discordant output) overlap in content with audit criteria three and four, so the higher concordance of the governance-engaged class is partly built into its definition. The class comparison is therefore descriptive and is not independent evidence for the literacy–concordance association.

### 3.9. Robustness, Exploratory Mediation, and the Illustrative Prediction Model

Table 6 brings together the robustness checks for the training–concordance association, the exploratory mediation decomposition, and the internal validation and decision-curve results for the illustrative prediction model. The complete specification of the illustrative prediction model is available from the corresponding author on request.

The training association was attenuated and no longer statistically significant in the inverse-probability-weighted analysis (OR 2.01, 95% CI 0.82–4.90; *p* = 0.126), in the post-hoc re-specified weighted analysis that included attitude towards AI (OR 1.84, 95% CI 0.71–4.77; *p* = 0.212), and in the extended-adjustment model (aOR 2.39, 95% CI 0.72–7.92; *p* = 0.155). Multiple imputation with the Model 1 covariates alone gave an estimate close to that of Model 1 (aOR 3.15, 95% CI 1.03–9.64; *p* = 0.044). Weighting left residual imbalance in nursing qualification (SMD 0.21). The E-value of 2.12 for the weighted estimate indicates that an unmeasured confounder associated with both training and concordance by risk ratios of about 2.1 could explain the point estimate. The E-value of 1.00 for the confidence limit reflects an interval that already includes the null. In the exploratory mediation decomposition, the estimated indirect component through AI legal literacy (0.278, 95% CI 0.133–0.429) was similar in size to the total association, and the direct component was close to zero (−0.036, 95% CI −0.215 to 0.164). This decomposition describes the structure of a cross-sectional association. Given the overlap between the training content and the literacy instrument, the unverifiable identifying assumptions, and the non-significant weighted estimates, it is consistent with, but does not demonstrate, an indirect pathway from training through literacy. The illustrative prediction model had an optimism-corrected AUC of 0.830 and a bootstrap-corrected calibration slope of 0.72, indicating appreciable overfitting. Its bootstrap-corrected net benefit exceeded that of remediating every nurse across the pre-specified 20–33% range of threshold probabilities, but by a smaller margin than the apparent net benefit (for example, 84 rather than 138 fewer unnecessary remediations per 1000 nurses at a 20% threshold). The literacy-only model performed almost as well (optimism-corrected AUC 0.812; Table 6). These estimates come from an internally evaluated model developed in a sample below the recommended minimum and are not evidence of clinical utility.

### 3.10. Multilevel Confirmation of the Primary Association

Table 7 reports the multilevel analyses that use the full hierarchy of interactions within nurses within wards.

Interaction-level concordance was observed in 661 of the 870 audited interactions (76.0%). By construction, all 440 interactions of the 44 concordant nurses were concordant, as were 221 of the 430 interactions (51.4%) of non-concordant nurses. Variation in interaction-level concordance was located predominantly at the nurse level (random-intercept variance 4.12; ICC 0.57), whereas the ward-level variance was small and not statistically significant (0.21; ICC 0.03; likelihood-ratio *p* = 0.412). In the three-level model, each 10-point increase in AI legal literacy was associated with higher odds of interaction-level concordance (aOR 3.61, 95% CI 1.98–6.58; *p* < 0.001). Prior training was not independently associated with concordance (aOR 1.09, 95% CI 0.36–3.28). The population-averaged estimate from generalised estimating equations was, as expected, somewhat closer to the null (OR 3.02, 95% CI 1.79–5.10). At the nurse level, adding a ward random intercept to Model 2 or using ward-clustered robust standard errors left the literacy estimate essentially unchanged (aOR 5.09 and 5.16, respectively), with the ward variance estimated near zero (Table 7). When interaction-level concordance was redefined using only the four record-based criteria, the literacy estimate was unchanged (aOR 3.44 per 10 points, 95% CI 1.90–6.22; *p* < 0.001). In summary, the multilevel models confirm the primary finding: neither the hierarchical structure of the data nor the use of a nurse-level binary outcome altered the inference.

## 4. Discussion

### 4.1. Analysis of Findings

The principal finding was a strong association between AI legal literacy and procedure-concordant use of the AI-enabled early-warning system. Prior governance training was associated with concordant practice in crude and initial adjusted analyses, but this association was attenuated after legal literacy was entered into the multivariable model, which is expected when a potential mediator is conditioned on, was no longer statistically significant after inverse-probability weighting, and in an exploratory decomposition, the indirect component through literacy was similar in size to the total association. The data therefore show attenuation of the training association once literacy is accounted for and an exploratory indirect association; they do not establish mediation in a causal sense. The association was consistent when the full hierarchical structure of the data was modelled, with three-level mixed-effects models of 870 audited interactions locating almost all of the variation at the nurse rather than the ward level. This pattern is compatible with, although it cannot establish, the hypothesis that attendance at training matters less than the legal literacy retained and applied at the bedside. The low awareness of specific legal instruments observed in the cohort is consistent with nursing studies showing that staff may receive data-protection instruction without developing a detailed understanding of the obligations governing clinical data processing [26]. It also aligns with concerns that transparency requirements have limited practical value unless clinicians can translate them into defensible clinical and documentation decisions [27]. Evidence of algorithmic bias [28] and work on informed consent for AI-assisted care [29] further support the need for clinicians to understand when human review, disclosure, escalation, and documentation are required. The absence of an association between alert volume and concordant documentation is also compatible with evidence that repeated exposure to decision-support alerts does not necessarily improve response quality [30].

Two conceptual points qualify the principal finding. First, the association demonstrated here is with the measured score, which captures factual legal knowledge and the ability to identify what a rule requires in a described scenario; it does not capture the ability to act on that interpretation under the time pressure of a real alert. The study therefore shows an association between the knowledge and applied-interpretation components of AI legal literacy (Section 1) and documented behaviour, and the broader claim that legal literacy in its full sense drives practice remains to be tested with performance-based measures. Second, the five domains of the instrument and the five audit criteria were derived from the same legal and institutional sources, so a nurse who knows that the SOP requires the tool version to be recorded is being tested on knowledge of the very behaviour that is audited. This overlap does not invalidate the association, because knowing a requirement and documenting it in routine care are different things, and the audit measured documented practice over 10 interactions rather than self-report. It does mean, however, that part of the large effect size may reflect shared content between the measure and the outcome, and that the association may be partly explained by general conscientiousness, competence, or educational attainment, which adjustment for qualification addresses only in part. The magnitude of the association should be read with these points in mind.

The findings can be placed alongside empirical nursing work on AI use, documentation, and data protection. Qualitative work with nurses using AI-enabled decision support from triage to intensive care points to the need for clear local guidance on responsibility and documentation when outputs are followed or overridden [31]. A systematic review of AI in nursing and midwifery found that most studies evaluate algorithm performance and few examine how nurses document or account for AI-informed decisions [32], and an international think tank identified governance, accountability, and education as priorities for the profession [33]. Surveys of healthcare staff show favourable perceptions of AI alongside limited knowledge of its implications [34]. Nursing documentation quality is typically assessed against explicit criteria for structure, completeness, and accuracy [35], and it improves when explicit standards are combined with audit and feedback [36]. The present study adds audited behavioural evidence to this literature and suggests that, in this sample, measured AI legal literacy was more closely aligned with how nurses documented AI-assisted care than was attendance at training or favourable attitudes. This alignment should be read with the caveat that the literacy instrument and the audit criteria share content (Section 4.2), which may account for part of the association.

The findings also highlight the organisational dimension of lawful AI use. Awareness of the institutional standard operating procedure was associated with more concordant behaviour, whereas alert volume itself was not; periodic audit feedback showed a positive but imprecise and non-significant association (OR 1.56, 95% CI 0.66–3.69), and the higher concordance of the governance-engaged latent class is partly built into its definition because two of its indicators overlap with the audit criteria. Similar documentation-improvement programmes have been most effective when explicit standards are combined with audit and feedback [36], and qualitative studies of healthcare leaders identify competence gaps, unclear accountability, and absent local guidance as recurrent barriers to AI implementation [37]. Educational interventions can improve knowledge rather than confidence alone [38], while governance frameworks emphasise named oversight roles and clear escalation pathways [39]. The persistence of incomplete human-verification documentation in the present study is also relevant because incorrect algorithmic advice can impair expert judgement through automation bias [40]. Together, these observations support governance strategies that integrate formal teaching with operational procedures, audit feedback, and visible accountability.

From a clinical and policy perspective, and if these associations are confirmed prospectively, literacy programmes should extend beyond general awareness of AI to the legal and operational decisions nurses make during routine care: identifying the system in use, understanding the applicable regulatory status, documenting human oversight, communicating AI involvement when required, protecting patient data, and reporting discordant outputs. Training should also address cybersecurity and model manipulation, because governance failures may arise from both inappropriate use and technical compromise [41]. Evaluation of such programmes should follow rigorous reporting principles for AI interventions [23,42] and should assess calibration and net benefit when predictive models are used to target remediation [25,43]. In the present study, an illustrative model based largely on the literacy score offered a higher bootstrap-corrected net benefit than remediating every nurse across the threshold probabilities that the hospital’s governance committee considered plausible (20–33%), but its sample size was well below the minimum recommended for model development [24], its bootstrap-corrected calibration slope of 0.72 indicates substantial overfitting, and a literacy-only model performed almost as well; the model is therefore a hypothesis about how remediation might be targeted between audit cycles, not a tool ready for deployment, and its apparent utility may not be reproducible in an independent population. This is particularly important as European health professionals are being asked to acquire AI-related competencies, while regulatory requirements and clinical curricula are evolving in parallel [44,45].

Future research should test these findings prospectively across multiple hospitals and nursing specialties. Longitudinal or controlled intervention studies are needed to determine whether literacy-focused education produces durable changes in documentation and governance behaviour rather than short-term knowledge gains; the present study did not evaluate an educational intervention, and its findings support the case for such evaluation rather than showing that education improves practice. The legal-literacy instrument should be externally validated, and future studies should examine whether improvements in concordant AI use are associated with patient-safety outcomes, incident reporting, trust, and workflow burden. External validation of the predictive model is also required before it is used to target individual staff for remediation, with particular attention to transportability across institutions, AI systems, and changing regulatory requirements [42,43,45].

### 4.2. Study Limitations

This study should be interpreted in light of several limitations. First, its cross-sectional design does not establish temporal ordering or causality; participants were unaware of the study during the audit window, which limits Hawthorne effects on the audited behaviour, but literacy was measured after that window, so it cannot be claimed that literacy preceded behaviour. The mediation analysis is therefore exploratory, its identifying assumptions cannot be verified, the training association was not statistically significant after inverse-probability weighting or multiple imputation, and weighting did not achieve complete covariate balance (residual SMD 0.21 for nursing qualification). The decomposition describes a pattern compatible with the observed data but cannot prove that training preceded and caused changes in literacy or documentation behaviour. Second, the study was conducted in a single tertiary centre with a modest sample, resulting in imprecise subgroup and interaction estimates and limiting external generalisability; with only 16 wards, ward-level variance components are estimated imprecisely, and the small ward-level variance observed here should not be taken to exclude clustering in other settings. The outcome is concordance with one hospital’s SOP, which operationalises the AI Act, the GDPR, and Romanian law in a particular way; institutional governance arrangements, the professional responsibilities of nurses, training structures, and the pace of implementation of EU requirements differ between hospitals and countries, so neither the prevalence of concordance nor the size of the association should be assumed to transfer directly to other settings. The sample was also below the minimum recommended for developing a prediction model, so the literacy-based model is exploratory, and its coefficients are likely to be over-fitted. Third, training exposure was observational rather than randomised, so residual and unmeasured confounding may remain despite multivariable adjustment, inverse-probability weighting, multiple imputation, and sensitivity analyses. Fourth, several measures, including attitudes, self-efficacy, perceived legal risk, and organisational support, were self-reported and may be affected by recall or response bias. The legal-literacy instrument was developed for this study; although content validation, pilot testing, item analysis, and a preliminary dimensionality assessment were performed, it has no external validation, its construct validity rests on known-groups differences, and it should be regarded as preliminary. The training content, the literacy instrument, and the audit criteria also derive from the same regulatory sources, so the attenuation of the training association after adjustment for literacy is partly expected by design (Section 2.4), and shared content may inflate the literacy–concordance association. Fifth, the documentation audit reflected the institutional AI-EWS workflow and local standard operating procedure; it may not capture every dimension of lawful AI use or apply directly to other AI systems. Two criteria were conditional on a triggering event, and nurses without such an event were classified as satisfying them; although the sensitivity analyses restricted to nurses with applicable interactions and using only unconditional criteria gave similar results, the number of triggering events per nurse was small, and the classification of conditional criteria remains a source of misclassification. The data-transfer criterion relied on institutional monitoring logs and note content and therefore captures only detected transfers from hospital systems; transfers from personal devices could not be observed, so non-compliance with this criterion is probably underestimated. Sixth, although the study used only a binary indicator already generated by routine institutional monitoring, and participants consented explicitly to its use, research use of employee-monitoring data can create perceived pressure to participate; the high participation rate is reassuring with respect to selection bias but should be read with this in mind. Seventh, a small amount of data was missing for selected covariates, and potentially relevant factors such as workload at the moment of an alert, ward culture, physician behaviour, and AI-EWS accuracy were not measured. Finally, the number of analyses performed is large relative to the sample; the analyses have therefore been organised into primary, secondary, and exploratory tiers (Section 2.4), and the exploratory results should not be given weight comparable to the primary association. These limitations support cautious interpretation and the need for multicentre prospective validation.

## 5. Conclusions

Greater AI legal literacy was strongly associated with procedure-concordant use of an AI-enabled early-warning system among hospital nurses in a single Romanian centre. Prior governance training was associated with concordant practice in unadjusted analyses, but this association was attenuated after adjustment for literacy and was not statistically significant after weighting; the cross-sectional design cannot determine whether training produced literacy or whether literacy produced concordant behaviour, and no causal pathway from training through literacy is claimed. These findings support the prospective evaluation of practice-oriented education on AI regulation, human oversight, documentation, data protection, patient information, and incident reporting, reinforced by clear institutional procedures and audit feedback. Multicentre prospective studies should confirm these relationships, validate the legal-literacy instrument, and determine whether literacy-focused interventions lead to sustained improvements in safe and legally concordant clinical AI use. The literacy-based model for targeting remediation is illustrative and hypothesis-generating and requires external validation before any use.

## Figures and Tables

**Figure 1:**
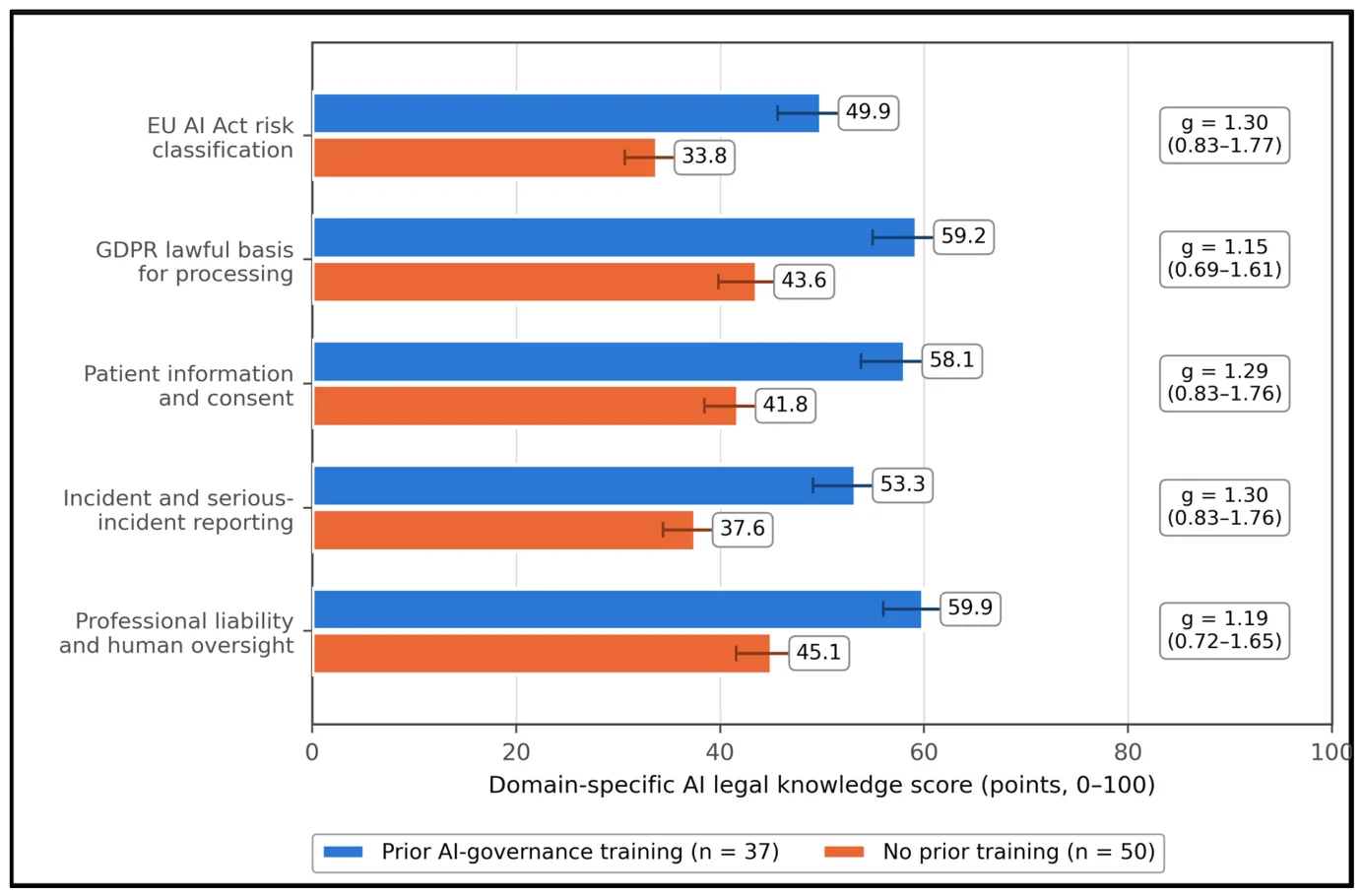
Domain-specific artificial intelligence (AI) legal literacy scores among 87 nurses, according to prior AI-governance training. Bars show group means with 95% confidence intervals; labels give the mean score and the standardised between-group difference (Hedges’ g with 95% confidence interval). AI, artificial intelligence; GDPR, General Data Protection Regulation; g, Hedges’ g.

**Figure 2:**
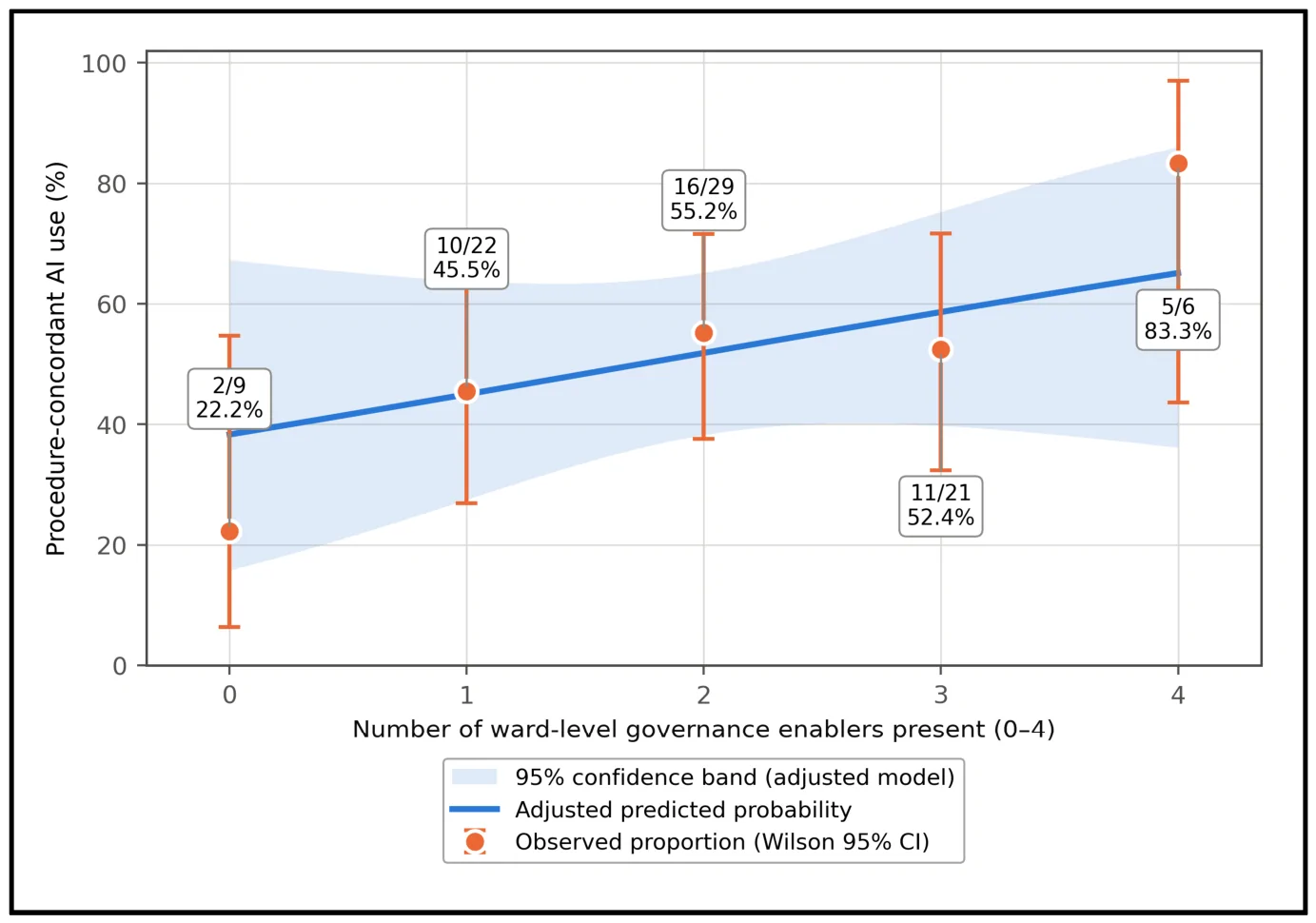
Dose–response relationship between the number of ward-level governance enablers present and procedure-concordant artificial intelligence (AI) use. Points show observed proportions with Wilson 95% confidence intervals; the line and shaded band show the predicted probability and 95% confidence band from a logistic model adjusted for AI legal literacy, prior training, age, sex, nursing qualification and clinical area (Model 2; Section 3.7). AI, artificial intelligence; CI, confidence interval.

**Figure 3:**
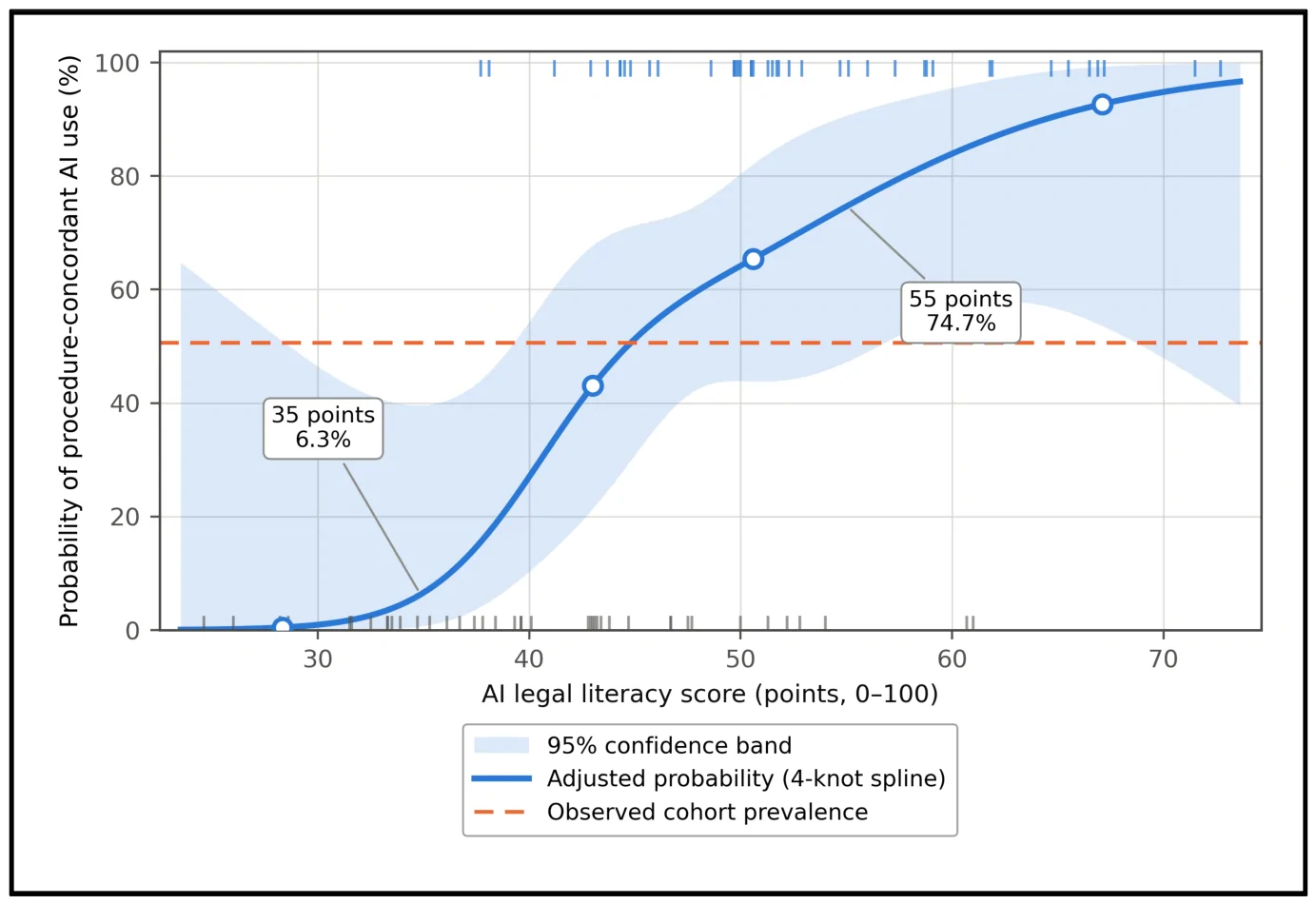
Restricted cubic spline model of the adjusted probability of procedure-concordant artificial intelligence (AI) use across the AI legal literacy score. The spline uses four knots at the 5th, 35th, 65th, and 95th centiles and is adjusted for prior AI-governance training, age, sex, nursing qualification, clinical area, and ward governance enablers; open circles mark knot positions and the blue (upper) and grey (lower) vertical rug marks show the observed distribution of literacy scores for concordant and non-concordant nurses, respectively; the dashed horizontal line marks the observed cohort prevalence of concordant use (50.6%). AI, artificial intelligence; CI, confidence interval.

**Table 1:** Baseline characteristics of the 87 nursing staff, overall and by prior artificial intelligence-governance training.

Characteristic	Overall (*n* = 87)	Prior AI-Governance Training (*n* = 37)	No Prior Training (*n* = 50)	SMD	*p*-Value
Age, years	38.7 ± 9.9	37.9 ± 9.9	39.2 ± 10.0	−0.14	0.440
Female sex, *n* (%)	72 (82.8)	28 (75.7)	44 (88.0)	−0.32	0.158
Nursing experience, years	12.8 ± 8.2	12.1 ± 7.9	13.3 ± 8.5	−0.15	0.531
**Highest nursing qualification, *n* (%)**				0.85	0.002
Post-secondary nursing school	30 (34.5)	5 (13.5)	25 (50.0)		
Bachelor of nursing	35 (40.2)	20 (54.1)	15 (30.0)		
Master of nursing	22 (25.3)	12 (32.4)	10 (20.0)		
**Clinical area, *n* (%)**				0.21	0.848
Intensive care	19 (21.8)	9 (24.3)	10 (20.0)		
Emergency	24 (27.6)	11 (29.7)	13 (26.0)		
Surgical	18 (20.7)	6 (16.2)	12 (24.0)		
Medical	26 (29.9)	11 (29.7)	15 (30.0)		
Charge/senior nurse, *n* (%)	18 (20.7)	11 (29.7)	7 (14.0)	0.39	0.108
Night shifts per month	6.1 ± 2.2	5.9 ± 2.1	6.3 ± 2.3	−0.21	0.343
Patients per shift	8.7 ± 3.8	8.6 ± 4.2	8.8 ± 3.6	−0.05	0.982
English proficiency ≥ B2, *n* (%)	41 (47.1)	18 (48.6)	23 (46.0)	0.05	0.831
Ward governance enablers, mean (0–4)	1.9 ± 1.1	2.2 ± 0.9	1.7 ± 1.2	0.49	0.037
Receives periodic audit feedback, *n* (%)	35 (40.2)	20 (54.1)	15 (30.0)	0.50	0.029
Digital self-efficacy, 0–100	53.6 ± 13.3	57.6 ± 11.2	50.7 ± 14.1	0.54	0.013
Attitude towards clinical AI, 0–100	50.8 ± 11.5	57.3 ± 8.1	46.0 ± 11.3	1.15	<0.001
Perceived organisational support, 0–100	58.4 ± 10.9	61.5 ± 9.7	56.0 ± 11.3	0.52	0.020
AI-EWS alerts reviewed per shift	4.3 ± 2.1	4.7 ± 2.2	4.1 ± 2.0	0.28	0.233

Continuous variables are presented as mean ± standard deviation and categorical variables as *n* (%). Continuous variables were compared with Welch’s *t* test when both groups satisfied the Shapiro–Wilk normality criterion (*p* > 0.05) and with the Mann–Whitney U test otherwise. All 2 × 2 categorical comparisons used Fisher’s exact test and larger contingency tables the Fisher–Freeman–Halton exact test with a Monte Carlo *p*-value (20,000 replicates). Bold type indicates category subheadings. Ward governance enablers are the count (0–4) of a designated AI champion, a visible standard operating procedure, superuser training, and periodic audit feedback. Missing values: patients per shift *n* = 3, night shifts per month *n* = 2, perceived organisational support *n* = 4. Abbreviations: AI, artificial intelligence; AI-EWS, artificial-intelligence-enabled early-warning system; B2, Common European Framework of Reference level B2; *n*, number of participants; SMD, standardised mean difference.

**Table 2:** Primary and secondary outcomes, overall and by prior artificial intelligence-governance training.

Outcome	Overall (*n* = 87)	Prior AI-Governance Training (*n* = 37)	No Prior Training (*n* = 50)	Effect Estimate (95% CI)	*p*-Value
Procedure-concordant AI use (all 5 criteria), *n* (%)	44 (50.6)	26 (70.3)	18 (36.0)	RD 34.3% (14.4 to 54.1); RR 1.95 (1.28 to 2.99); OR 4.20 (1.69 to 10.45)	0.002
Audit criteria met, *n* (0–5)	4.1 ± 1.2	4.5 ± 0.9	3.7 ± 1.2	MD 0.8 (0.3 to 1.3); g 0.72 (0.28 to 1.16)	<0.001
AI legal literacy score, 0–100	47.1 ± 12.1	55.5 ± 10.7	40.8 ± 9.0	MD 14.7 (10.4 to 19.1); g 1.50 (1.02 to 1.99)	<0.001
Attitude towards clinical AI, 0–100	50.8 ± 11.5	57.3 ± 8.1	46.0 ± 11.3	MD 11.3 (7.1 to 15.4); g 1.11 (0.65 to 1.57)	<0.001
Perceived legal risk, 0–100	64.1 ± 12.1	58.8 ± 10.6	68.1 ± 11.7	MD −9.3 (−14.1 to −4.5); g −0.82 (−1.26 to −0.38)	<0.001
Digital self-efficacy, 0–100	53.6 ± 13.3	57.6 ± 11.2	50.7 ± 14.1	MD 6.8 (1.5 to 12.2); g 0.52 (0.09 to 0.96)	0.013
Aware of Regulation (EU) 2024/1689, *n* (%)	26 (29.9)	15 (40.5)	11 (22.0)	RD 18.5% (−1.0 to 38.1); RR 1.84 (0.96 to 3.54); OR 2.42 (0.95 to 6.17)	0.096
Aware of institutional AI standard operating procedure, *n* (%)	49 (56.3)	27 (73.0)	22 (44.0)	RD 29.0% (9.1 to 48.8); RR 1.66 (1.15 to 2.40); OR 3.44 (1.38 to 8.58)	0.009
Routine consent notation, *n* (%)	35 (40.2)	21 (56.8)	14 (28.0)	RD 28.8% (8.5 to 49.0); RR 2.03 (1.20 to 3.43); OR 3.38 (1.38 to 8.27)	0.009
Routine reporting of discordant output, *n* (%)	54 (62.1)	31 (83.8)	23 (46.0)	RD 37.8% (19.6 to 56.0); RR 1.82 (1.31 to 2.54); OR 6.07 (2.15 to 17.09)	<0.001

Binary outcomes are *n* (%); continuous outcomes are mean ± standard deviation. Effect estimates for binary outcomes are the risk difference, risk ratio and odds ratio with 95% confidence intervals, and *p*-values are from Fisher’s exact test. Effect estimates for continuous outcomes are the mean difference (Welch 95% confidence interval) and Hedges’ g, with *p*-values from Welch’s *t* test or the Mann–Whitney U test according to the Shapiro–Wilk criterion. Procedure-concordant AI use required all five documentation-audit criteria to be satisfied. Abbreviations: AI, artificial intelligence; CI, confidence interval; g, Hedges’ g; MD, mean difference; OR, odds ratio; RD, risk difference; RR, risk ratio; *n*, number of participants.

**Table 3:** Documentation-audit criteria, procedure-concordant use, and AI legal literacy measures across the four clinical areas.

Variable	Intensive Care (*n* = 19)	Emergency (*n* = 24)	Surgical (*n* = 18)	Medical (*n* = 26)	Overall (*n* = 87)	Effect Size	*p*-Value
Human verification of AI output documented, *n* (%)	17 (89.5)	22 (91.7)	13 (72.2)	22 (84.6)	74 (85.1)	0.20	0.334
AI tool identifier and version recorded, *n* (%)	14 (73.7)	23 (95.8)	8 (44.4)	21 (80.8)	66 (75.9)	0.42	0.001
Patient information or consent noted, *n* (%)	15 (78.9)	19 (79.2)	15 (83.3)	21 (80.8)	70 (80.5)	0.04	1.000
Discordant AI output reported via incident system, *n* (%)	14 (73.7)	18 (75.0)	13 (72.2)	19 (73.1)	64 (73.6)	0.02	1.000
No transfer of data to unapproved AI tools, *n* (%)	19 (100.0)	23 (95.8)	14 (77.8)	25 (96.2)	81 (93.1)	0.32	0.041
Procedure-concordant AI use (all five criteria), *n* (%)	8 (42.1)	14 (58.3)	6 (33.3)	16 (61.5)	44 (50.6)	0.23	0.215
Audit criteria met, mean (0–5)	4.2 ± 0.9	4.4 ± 0.9	3.5 ± 1.4	4.2 ± 1.3	4.1 ± 1.2	ε^2^ 0.031	0.135
AI legal literacy score, 0–100	47.0 ± 12.8	49.6 ± 10.6	42.4 ± 9.5	48.0 ± 14.2	47.1 ± 12.1	η^2^ 0.045	0.284
Perceived legal risk, 0–100	62.9 ± 13.3	62.6 ± 12.4	66.9 ± 9.8	64.4 ± 12.8	64.1 ± 12.1	ε^2^ −0.015	0.633
Ward governance enablers, mean (0–4)	1.9 ± 1.2	2.2 ± 1.0	1.6 ± 1.1	1.9 ± 1.1	1.9 ± 1.1	ε^2^ 0.017	0.218

Categorical variables are *n* (%) and were compared with the Fisher–Freeman–Halton exact test (Monte Carlo *p*-value, 20,000 replicates), with Cramér’s V as the effect size. Continuous variables are mean ± standard deviation and were compared with one-way analysis of variance (effect size η^2^) when the Shapiro–Wilk and Levene criteria were satisfied and with the Kruskal–Wallis test (effect size ε^2^) otherwise; a negative ε^2^ indicates a test statistic smaller than its degrees of freedom and is interpreted as zero. Post-hoc pairwise comparisons used Dunn’s test with Holm adjustment. Abbreviations: AI, artificial intelligence; *n*, number of participants; V, Cramér’s V; ε^2^, epsilon-squared; η^2^, eta-squared.

**Table 4:** Univariable associations with procedure-concordant artificial intelligence use.

Variable	*n*	Unadjusted OR	95% CI	*p*-Value	q-Value
Age, per year	87	0.96	0.92–1.00	0.061	0.098
Female sex	87	1.68	0.54–5.20	0.371	0.433
Nursing experience, per year	87	0.95	0.90–1.00	0.053	0.092
Bachelor or Master qualification	87	4.71	1.78–12.46	0.002	0.009
Charge/senior nurse	87	1.71	0.59–4.94	0.318	0.393
Intensive care area	87	0.65	0.23–1.81	0.405	0.448
Night shifts per month, per shift	85	1.00	0.83–1.21	0.986	0.986
Patients per shift, per patient	84	1.07	0.95–1.20	0.246	0.344
English proficiency ≥ B2	87	2.22	0.94–5.24	0.069	0.103
Prior AI-governance training	87	4.20	1.69–10.45	0.002	0.009
AI legal literacy, per 10 points	87	5.47	2.59–11.56	<0.001	<0.001
Attitude towards clinical AI, per 10 points	87	1.97	1.26–3.09	0.003	0.011
Digital self-efficacy, per 10 points	87	1.57	1.10–2.25	0.013	0.034
Perceived legal risk, per 10 points	87	0.60	0.40–0.89	0.011	0.033
Perceived organisational support, per 10 points	83	1.55	1.01–2.37	0.045	0.085
Ward governance enablers, per enabler	87	1.53	1.02–2.32	0.041	0.085
Periodic audit feedback	87	1.56	0.66–3.69	0.316	0.393
Aware of Regulation (EU) 2024/1689	87	5.14	1.80–14.64	0.002	0.009
Aware of institutional AI SOP	87	4.08	1.65–10.05	0.002	0.009
Regular use of AI-enabled tools	87	3.93	1.28–12.07	0.017	0.039
AI-EWS alerts reviewed per shift, per alert	87	0.93	0.75–1.14	0.460	0.483

Each row is a separate univariable logistic regression with procedure-concordant AI use as the dependent variable; *n* is the number of participants contributing to that model after listwise deletion. q-values are Benjamini–Hochberg false-discovery-rate-adjusted *p*-values across all 21 tests. Continuous psychometric scales are scaled per 10 points. Abbreviations: AI, artificial intelligence; AI-EWS, artificial-intelligence-enabled early-warning system; B2, Common European Framework of Reference level B2; CI, confidence interval; *n*, number of participants; OR, odds ratio; SOP, standard operating procedure.

**Table 5:** Multivariable logistic regression models for procedure-concordant artificial intelligence use.

Predictor	Model 1 aOR (95% CI)	*p*-Value	Model 2 aOR (95% CI)	*p*-Value	VIF
Prior AI-governance training (yes vs. no)	3.22 (1.06–9.81)	0.039	0.74 (0.18–3.06)	0.683	1.82
Age, per year	0.97 (0.92–1.03)	0.318	0.98 (0.92–1.04)	0.470	1.25
Female sex (vs. male)	3.55 (0.89–14.22)	0.073	2.12 (0.44–10.18)	0.347	1.15
Bachelor or Master qualification (vs. post-secondary)	3.70 (1.10–12.48)	0.035	1.28 (0.33–5.00)	0.726	1.64
Intensive care area (vs. other wards)	0.52 (0.16–1.70)	0.280	0.57 (0.13–2.45)	0.451	1.01
Ward governance enablers, per enabler	1.42 (0.87–2.31)	0.157	1.32 (0.77–2.24)	0.309	1.14
AI legal literacy, per 10 points	—	—	5.16 (2.04–13.06)	<0.001	2.00

Model 1 adjusts for age, sex, nursing qualification, clinical area and ward governance enablers; Model 2 adds the AI legal literacy score. Both models were fitted to all 87 participants with procedure-concordant AI use as the dependent variable. Model 1: likelihood-ratio χ^2^ = 24.04 on 6 degrees of freedom, *p* < 0.001; McFadden pseudo-R^2^ = 0.199; AIC = 110.6; area under the curve = 0.787; Hosmer–Lemeshow χ^2^ = 5.18 on 8 degrees of freedom, *p* = 0.738; Brier score = 0.189. Model 2: likelihood-ratio χ^2^ = 43.23 on 7 degrees of freedom, *p* < 0.001; McFadden pseudo-R^2^ = 0.358; AIC = 93.4; area under the curve = 0.875; Hosmer–Lemeshow χ^2^ = 8.89 on 8 degrees of freedom, *p* = 0.352; Brier score = 0.144. Variance inflation factors are from Model 2. Hosmer–Lemeshow statistics for both models use ten groups of predicted risk, the same specification as Table 6. Abbreviations: AI, artificial intelligence; AIC, Akaike information criterion; aOR, adjusted odds ratio; CI, confidence interval; VIF, variance inflation factor.

**Table 6:** Robustness analyses of the training association, exploratory mediation decomposition, and internal validation of the illustrative prediction model.

Analysis and Parameter	Estimate	95% CI	*p*-Value
**Panel A. Robustness of the association between prior AI-governance training and procedure-concordant AI use**			
Crude odds ratio	4.20	1.69–10.45	0.002
Multivariable adjusted odds ratio	3.22	1.06–9.81	0.039
Stabilised IPTW odds ratio	2.01	0.82–4.90	0.126
Stabilised IPTW risk difference, %	17.2	−4.5 to 40.8	—
Stabilised IPTW odds ratio, re-specified propensity model (post hoc)	1.84	0.71–4.77	0.212
Multiple-imputation adjusted odds ratio (Model 1 covariates)	3.15	1.03–9.64	0.044
Extended-adjustment model (multiple imputation, adding perceived organisational support and perceived legal risk)	2.39	0.72–7.92	0.155
E-value for the IPTW estimate (weighted risk ratio 1.39)	2.12	—	—
E-value for the confidence limit closest to the null	1.00	—	—
Maximum absolute SMD after weighting (nursing qualification)	0.21	—	—
**Panel B. Exploratory mediation decomposition (non-causal): training → AI legal literacy → concordant use**			
a-path: literacy per 10 points, per training	1.224	0.790–1.658	<0.001
b-path: log-odds per 10 literacy points	1.641	0.712–2.570	<0.001
Indirect effect through literacy (ACME)	0.278	0.133–0.429	<0.001
Average direct effect (ADE)	−0.036	−0.215 to 0.164	0.686
Total effect	0.242	0.024–0.454	0.028
**Panel C. Internal validation and decision-curve analysis of the illustrative prediction model (hypothesis-generating)**			
Apparent area under the curve	0.875	—	—
Bootstrap optimism	0.044	—	—
Optimism-corrected area under the curve	0.830	0.761–0.904	—
Calibration intercept	0.00	—	—
Apparent calibration slope	1.00 (by definition)	—	—
Bootstrap-corrected calibration slope	0.72	—	—
Brier score	0.144	—	—
Scaled Brier score	0.425	—	—
Hosmer–Lemeshow test (8 df)	χ^2^ = 8.89	—	0.352
Net benefit at a 20% threshold of non-concordance (model vs. remediate-all)	0.402 vs. 0.368 (corrected 0.389 vs. 0.368)	Δ 0.034 (corrected 0.021)	138 (corrected 84) per 1000 ᵇ
Net benefit at a 25% threshold of non-concordance (model vs. remediate-all)	0.372 vs. 0.325 (corrected 0.358 vs. 0.325)	Δ 0.047 (corrected 0.033)	140 (corrected 99) per 1000 ᵇ
Net benefit at a 33% threshold of non-concordance (model vs. remediate-all)	0.351 vs. 0.245 (corrected 0.329 vs. 0.245)	Δ 0.106 (corrected 0.084)	215 (corrected 171) per 1000 ᵇ
Literacy-only model: optimism-corrected area under the curve	0.812	0.738–0.886	—
Literacy-only model: bootstrap-corrected calibration slope	0.81	—	—

Panel A: the multivariable model adjusts for age, sex, nursing qualification, clinical area and ward governance enablers; stabilised inverse-probability-of-treatment weights were derived from a propensity model including age, sex, qualification, intensive care assignment, ward governance enablers, English proficiency and digital self-efficacy (c-statistic 0.819) and truncated at the first and 99th percentiles (weight range 0.47–2.69); the maximum standardised mean difference after weighting was 0.21 (nursing qualification), all others ≤0.15, so that complete balance was not achieved and the weighted estimates should be interpreted with this residual imbalance in mind. The re-specified propensity model (post hoc) removed digital self-efficacy and added attitude towards AI and nursing experience (c-statistic 0.842; weight range 0.39–3.12; maximum standardised mean difference after weighting 0.18, attitude towards AI). Multiple imputation used chained equations with 20 imputations and Rubin’s rules (fraction of missing information < 0.001); it is reported with the Model 1 covariates only (missing-data sensitivity analysis) and, separately, as an extended-adjustment model that adds perceived organisational support and perceived legal risk and is therefore a different model rather than a missing-data analysis. The E-value was computed from the inverse-probability-weighted risk ratio (1.39) as RR + √[RR(RR − 1)]; because the weighted confidence interval includes the null, the E-value for the confidence limit is 1.00. Panel B: exploratory decomposition of the cross-sectional association using the quasi-Bayesian Monte Carlo approximation with 5000 simulations; ACME, ADE, and total effects are expressed as absolute differences in the probability of procedure-concordant use. The estimates rest on unverifiable no-unmeasured-confounding and temporal-ordering assumptions in this cross-sectional design and do not establish a causal mechanism. The proportion mediated is not reported because the direct-effect estimate was negative and close to zero, which makes the ratio unstable (point estimate > 1 with a very wide interval) and uninterpretable. Panel C: the final model is Model 2 of Table 5; optimism was estimated by 1000 bootstrap replications; the 95% confidence interval for the optimism-corrected area under the curve is the 2.5th–97.5th percentile range of the bootstrap distribution of the apparent area shifted by the mean optimism, and the apparent calibration slope of a maximum-likelihood logistic model is 1.00 by definition; the Hosmer–Lemeshow test uses ten groups of predicted risk, identical to Table 5. Decision-curve analysis was performed on the predicted probability of non-concordant use (1 minus the predicted probability of concordance), so that ‘treat’ denotes offering the targeted remediation described in Section 2.5; threshold probabilities of 20–33% correspond to judging a missed remediation two to four times more harmful than an unnecessary one, and for the decision-curve rows the last column (ᵇ) gives the net reduction in unnecessary remediations per 1000 nurses screened compared with remediating every nurse, not a *p*-value. Values marked “corrected” are bootstrap-corrected (apparent value minus mean optimism over 1000 replicates). The literacy-only model uses the literacy score as its sole predictor. Bold type indicates panel headings. Abbreviations: ACME, average causal mediation effect; ADE, average direct effect; AI, artificial intelligence; CI, confidence interval; df, degrees of freedom; IPTW, inverse-probability-of-treatment weighting; SMD, standardised mean difference; Δ, difference in net benefit; χ^2^, chi-square statistic.

**Table 7:** Multilevel analyses accounting for audited interactions nested within nurses and nurses nested within wards.

Model and Parameter	Estimate	95% CI	*p*-Value
**Interaction-level outcome (870 interactions; 661 concordant, 76.0%): three-level mixed-effects logistic regression (interactions in nurses in wards)**			
AI legal literacy, per 10 points, aOR	3.61	1.98–6.58	<0.001
Prior AI-governance training, aOR	1.09	0.36–3.28	0.881
Bachelor or Master qualification, aOR	1.41	0.47–4.21	0.539
Ward governance enablers, per enabler, aOR	1.24	0.79–1.95	0.352
Nurse-level random-intercept variance	4.12	2.31–7.35	<0.001 ᵃ
Ward-level random-intercept variance	0.21	0.00–1.38	0.412 ᵃ
ICC, nurse level (latent scale)	0.57	0.44–0.69	—
ICC, ward level (latent scale)	0.03	0.00–0.15	—
**Interaction-level outcome: generalised estimating equations, exchangeable correlation within nurse, cluster-robust SE**			
AI legal literacy, per 10 points, population-averaged OR	3.02	1.79–5.10	<0.001
Prior AI-governance training, population-averaged OR	1.12	0.45–2.79	0.804
**Nurse-level outcome (87 nurses): Model 2 with random intercept for ward**			
AI legal literacy, per 10 points, aOR	5.09	2.01–12.9	<0.001
Prior AI-governance training, aOR	0.76	0.18–3.16	0.706
Ward-level random-intercept variance	0.04	0.00–0.92	0.711 ᵃ
**Nurse-level outcome: Model 2 with cluster-robust standard errors for ward (16 clusters)**			
AI legal literacy, per 10 points, aOR	5.16	2.23–11.9	<0.001
Prior AI-governance training, aOR	0.74	0.21–2.62	0.641

Interaction-level concordance denotes all five criteria met in a given audited interaction, with conditional criteria scored as met when not applicable (Section 2.3). All models include the Model 2 fixed effects (prior training, age, sex, nursing qualification, intensive-care area, ward governance enablers and AI legal literacy); only the estimates of principal interest are shown, and the full output is available from the corresponding author on request. Mixed-effects models were fitted by adaptive Gauss–Hermite quadrature (15 points); intraclass correlation coefficients (ICC) are calculated on the latent scale with the level-1 variance fixed at π^2^/3: nurse-level ICC = (σ^2^nurse + σ^2^ward)/(σ^2^nurse + σ^2^ward + π^2^/3), the correlation between two interactions of the same nurse, and ward-level ICC = σ^2^ward/(σ^2^nurse + σ^2^ward + π^2^/3). ᵃ *p*-value from the likelihood-ratio test of the random intercept against the model without that random effect (mixture of χ^2^ distributions); the 95% CI for the variance is a profile-likelihood interval. Abbreviations: AI, artificial intelligence; aOR, adjusted odds ratio; CI, confidence interval; ICC, intraclass correlation coefficient; OR, odds ratio; SE, standard error. Bold type indicates model subheadings.

## Data Availability

The data presented in this study are available from the corresponding author upon reasonable request; participant-level data are not publicly available due to privacy and ethical restrictions. The complete analysis code (Python and R scripts for data preparation, all models, bootstrap validation and decision-curve analysis) is also available from the corresponding author upon reasonable request.

## Public Involvement Statement

No public involvement in any aspect of this research.

## Guidelines and Standards Statement

This manuscript was drafted against the Strengthening the Reporting of Observational Studies in Epidemiology (STROBE) statement [19] for cross-sectional observational research; the exploratory prediction model is reported in accordance with the Transparent Reporting of a multivariable prediction model for Individual Prognosis Or Diagnosis + Artificial Intelligence (TRIPOD+AI) statement [23].
